# Teledentistry: Applications Across Dental Specialties: A Systematic Review and Meta-Analysis 

**DOI:** 10.30476/dentjods.2025.106873.2726

**Published:** 2026-09-01

**Authors:** Aryan Tabibnia, Pooya Omidi, Saba Amini, Sam Shahbod, Arezoo Alaee, Kimia Ghods

**Affiliations:** 1 Dental Student, Membership of Dental Material Research Center, Tehran Medical Sciences, Islamic Azad University, Tehran, Iran.; 2 Dept. of Oral Medicine, Membership of Dental Material Research Center, School of Dentistry, Islamic Azad University, Tehran Medical Branch, Tehran, Iran.; 3 Membership of Dental Material Research Center, Tehran Medical Sciences, Islamic Azad University, Tehran, Iran.

**Keywords:** Teledentistry, Dentistry, Remote Consultation, Telemonitoring, Artificial Intelligence

## Abstract

**Background:**

Teledentistry is the use of digital communication technologies to provide remote dental care, which offers innovative solutions to enhance access, efficiency, and patient outcomes.

**Purpose:**

This article evaluates the diagnostic accuracy of teledentistry compared to in-person gold-standard examinations, primarily in restorative dentistry.

**Materials and Method:**

A systematic search of literature was conducted on PubMed, Google Scholar, Embase, and Scopus databases, covering studies from 2015 and 2024. Due to methodological heterogeneity and limited comparable studies, meta-analysis was only feasible for the field of restorative dentistry, and other specialties were analyzed descriptively. Furthermore, the Newcastle-Ottawa Scale was used to assess bias, which manifested a moderate-to-low risk of bias.

**Results:**

The results of 4 studies in restorative dentistry demonstrated a moderate sensitivity of 77.2% (95% CI 62.5-87.4%) and a high specificity of 89.1% (95% CI 70.5-96.5%). While significant heterogeneity persisted (I² = 68% for sensitivity and 92% for specificity), sensitivity analyses confirmed robust pooled estimates unaffected by individual study removal. This was attributable to methodological variations in diagnostic thresholds and image capture protocols despite uniform smartphone photography. On the other hand, descriptive analysis of studies in other fields revealed variable diagnostic accuracy, with teledentistry showing promise in orthodontics and prosthodontics but limitations in endodontics.

**Conclusion:**

The findings suggest teledentistry as a useful diagnostic tool in restorative dentistry, though it is not yet a complete substitute for in-person examinations. However, the generalizability of these findings to other dental specialties remains low due to the lack of an adequate number of relevant studies. Moreover, insufficient data exist to support the positive role of artificial intelligence (AI) in combination with tele-dentistry. Therefore, future work should prioritize standardized AI-tele-dentistry validation and expand research across diverse specialties to strengthen evidence.

## Introduction

Teledentistry is an innovative field that harnesses electronic information, imaging, and communication technologies to deliver and manage dental services. The term itself, "teledentistry," was first introduced in 1997 by Cook, who defined it as the application of Videocon ferencing for remote diagnosis and consultation in healthcare. However, contemporary frameworks from the American Dental Association (ADA; 2022) expand this to include "all digital health modalities enabling remote diagnosis, treatment planning, monitoring, and education through real-time and asynchronous technologies" [ 1 - 2 ].

Globally, a persistent healthcare challenge is ensuring access to medical professionals for individuals in rural, remote, and border areas. In other words, several factors including low ratios of healthcare providers to populations, the high cost of dental care, inadequate infrastructure and dental facilities, long distances to centralized dental services, limited transportation options for accessing care, insufficient oral health awareness, geographical barriers, poverty, and cultural factors prevalent in rural settings underscore the critical need for remote care solutions [ 2 ].

Teledentistry has significantly expanded its scope over time, notably extending its reach into rural communities. A prime example of this expansion is the US Army’s Total Dental Access Project. This initiative project effectively used teledentistry to bring dental care to underserved remote and rural populations, with the dual aims of improving patient outcomes and enhancing dental education [ 2 - 4 ]. The program achieved a 63% reduction in patient wait times for specialty consultations (*p*< 0.01) and a 41% improvement in treatment completion rates compared to traditional referral systems. Notably, radiographic diagnostic concordance between remote and in-person evaluations reached 92.4% (κ= 0.86) for caries detection in a 2021 validation study (n=1,247 cases). These outcomes demonstrate teledentistry's capacity to effectively address dental issues regardless of geographical distance [ 1 , 3 , 5 ].

Beyond the well-documented dental need in rural areas with limited resources, urban centers also stand to benefit from teledentistry services, especially during epidemics. The COVID-19 pandemic, for instance, highlighted its value in facilitating remote patient assessments (teletriage) and ensuring continuity of care (telemonitoring) for individuals with suspected or confirmed infections [ 6 - 8 ].

Even though teledentistry has emerged as a critical tool for improving access to care in rural and underserved regions, its applications extend far beyond geographical barriers. This technology demonstrates transformative potential across various dental specialties. In restorative dentistry, teledentistry facilitates remote assessment of tooth cavities and digital treatment planning. Similarly, in orthodontics, it enables effective monitoring of aligner therapy through virtual check-ins. Additional applications include oral surgery (pre- and post-operative consultations), periodontology (remote tracking of periodontal health), endodontics (teleconsultations for diagnosis and follow-up), prosthodontics (digital impression evaluations), and pediatric dentistry (early caries detection and behavioral guidance) [ 1 , 5 ]. Despite growing research on teledentistry, significant heterogeneity exists in reported outcomes across studies, particularly regarding diagnostic accuracy. This inconsistency highlights the critical need for a systematic review and meta-analysis to establish evidence-based consensus, identify moderating variables, and clarify optimal implementation protocols [ 1 , 3 , 5 ].

While the advantages of teledentistry are clear, research has also identified limitations, particularly concerning its capacity for definitive diagnostic accuracy in treatment planning. The diagnostic accuracy of teledentistry is influenced by multiple interdependent factors, with imaging technology limitations emerging as the most significant barrier [ 6 ]. Current evidence suggests that the resolution and color fidelity of consumer-grade intraoral cameras account for approximately 40-60% of diagnostic discrepancies in early caries detection. Besides, data transmission quality constitutes the second most critical factor, with JPEG compression artifacts and bandwidth limitations reducing diagnostic information by 15-20% in radiographic interpretations. While operator skill variability remains a persistent challenge (affecting 25-30% of cases, particularly in pediatric and oral medicine applications), its impact is generally secondary to technological limitations [ 6 - 7 , 9 ]. These factors, along with legal barriers, privacy risks, and ethical concerns, raise important questions about teledentistry's overall performance and effectiveness across different dental specialties [ 10 ]. Hence, this text serves as the introduction to a scientific article that examines the advantages, disadvantages, and potential applications of teledentistry.

## Materials and Method

### Information sources and search strategy

This systematic review adhered to the PRISMA guidelines [ 11 ]. The search was limited to peer-reviewed journal articles published in English, excluding books, editorials, reviews, commentaries, dissertations, unpublished materials, and letters to the editor. Studies published between January 2020 and December 2024 were included to ensure the review reflected the most recent advancements and clinical evidence. PubMed, Google Scholar, Embase, and Scopus databases were used to identify relevant studies using variations of key terms. The key terms were carefully chosen to align with the study's topic and subsequently were refined by Boolean operators, which can be seen in Table 1. The Boolean operators "AND" and "OR" were used to combine different concepts and include synonyms, respectively. Moreover, MeSH terms were employed to enhance the precision and sensitivity of the search.

**Table 1:** The strategy for Boolean search

Database	Keywords	Number of Results
PubMed	("Tele dentistry"[Mesh] OR "Teledentistry"[Mesh] OR "Tele-dentistry"[Title/Abstract] OR "Tele dentistry" [Title/Abstract]) AND ("Dentistry"[Mesh] OR "Remote Consultation"[Mesh] OR "Teleconsultation"[Title/Abstract] OR "Telemedicine"[Mesh] OR "Telemonitoring"[Mesh] OR "Remote Monitoring"[Mesh] OR "Teleradiology"[Mesh] OR "Radiology"[Mesh] OR "Artificial Intelligence"[Mesh] OR "AI"[Title/Abstract] OR "Machine Learning"[Mesh] OR "Deep Learning"[Mesh] OR "AI Applications"[Title/Abstract] OR "Military Dentistry"[Mesh] OR "Geriatric Dentistry"[Mesh] OR "Dental Care for Aged"[Mesh] OR "Orthodontics"[Mesh] OR "Endodontics"[Mesh] OR "Periodontics"[Mesh] OR "Oral Surgical Procedures"[Mesh] OR "Maxillofacial Surgery"[Title/Abstract] OR "Pediatric Dentistry"[Mesh] OR "Pedodontics"[Title/Abstract] OR "Prosthodontics"[Mesh] OR "Oral Medicine"[Mesh] OR "Pathology, Oral"[Mesh] OR "Public Health Dentistry"[Mesh])	156
Google Scholar	("Tele dentistry" OR "Teledentistry" OR "Tele-dentistry") AND ("Dentistry" OR "Remote Consultation" OR "Teleconsultation" OR "Telemedicine" OR "Telemonitoring" OR "Remote Monitoring" OR "Teleradiology" OR "Radiology" OR "Artificial Intelligence" OR "AI" OR "Machine Learning" OR "Deep Learning" OR "AI Applications" OR "Military Dentistry" OR "Geriatric Dentistry" OR "Dental Care for Aged" OR "Orthodontics" OR "Endodontics" OR "Periodontics" OR "Oral Surgical Procedures" OR "Maxillofacial Surgery" OR "Pediatric Dentistry" OR "Pedodontics" OR "Prosthodontics" OR "Oral Medicine" OR "Pathology, Oral" OR "Public Health Dentistry")	282
Embase	('tele dentistry'/exp OR 'teledentistry'/exp OR 'tele-dentistry':ti,ab OR 'tele dentistry':ti,ab) AND ('dentistry'/exp OR 'remote consultation'/exp OR 'teleconsultation':ti,ab OR 'telemedicine'/exp OR 'telemonitoring'/exp OR 'remote monitoring'/exp OR 'teleradiology'/exp OR 'radiology'/exp OR 'artificial intelligence'/exp OR 'ai':ti,ab OR 'machine learning'/exp OR 'deep learning'/exp OR 'ai applications':ti,ab OR 'military dentistry'/exp OR 'geriatric dentistry'/exp OR 'dental care for aged'/exp OR 'orthodontics'/exp OR 'endodontics'/exp OR 'periodontics'/exp OR 'oral surgical procedures'/exp OR 'maxillofacial surgery':ti,ab OR 'pediatric dentistry'/exp OR 'pedodontics':ti,ab OR 'prosthodontics'/exp OR 'oral medicine'/exp OR 'oral pathology'/exp OR 'public health dentistry'/exp)	82
Scopus	(TITLE-ABS-KEY("tele dentistry" OR "teledentistry" OR "tele-dentistry")) AND (TITLE-ABS-KEY("dentistry" OR "remote consultation" OR "teleconsultation" OR "telemedicine" OR "telemonitoring" OR "remote monitoring" OR "teleradiology" OR "radiology" OR "artificial intelligence" OR "ai" OR "machine learning" OR "deep learning" OR "ai applications" OR "military dentistry" OR "geriatric dentistry" OR "dental care for aged" OR "orthodontics" OR "endodontics" OR "periodontics" OR "oral surgical procedures" OR "maxillofacial surgery" OR "pediatric dentistry" OR "pedodontics" OR "prosthodontics" OR "oral medicine" OR "oral pathology" OR "public health dentistry"))	42

While Google Scholar was utilized as one of the primary databases in this systematic review due to its broad coverage, it’s indexing of duplicate records and non-peer-reviewed content posed some challenges in the search process. To mitigate these limitations, duplicates were removed, and results were limited to the top 200 most relevant records per query. Titles and abstracts were then assessed to ensure the inclusion of only relevant and peer-reviewed studies, thereby enhancing the reliability of the review.

### Eligibility Criteria

The selection process included studies that focused on telehealth or teledentistry applications, were published in English with full-text availability, and directly compared teledentistry methods to visual inspections or established reference standards. Studies were excluded if they were unrelated to telehealth or tele-dentistry, were preliminary pilot investigations with small sample sizes or overlapping data, lacked sufficient methodological detail, did not provide full-text access, failed to use appropriate statistics, failed to define reference standards, or were available only as abstracts. 

Since the systematic review's primary objective was to assess teledentistry's application against conventional clinical standards or gold standard methods in each branch of dentistry, no limitations were imposed regarding research methodologies, participant demographics, or selection approaches. Accordingly, to address potential heterogeneity arising from this variation, subgroup analyses and sensitivity assessments were planned to explore the influence of these factors on the overall findings.

### Study selection and data extraction

Two reviewers (AT and KG) independently screened titles and abstracts to identify potentially eligible studies, followed by full-text reviews to confirm inclusion. When necessary data were absent; the study authors were contacted for clarification. Discrepancies were resolved through discussion, with a third researcher (PO) acting as an arbitrator when consensus could not be reached. 

Two reviewers (AT and KG) individually extracted data from the included studies using a standardized data collection form; Rayyan [ 12 ]. Extracted data included study details such as first author, publication year, country of origin, research methodology, participant characteristics (sample size, age range, procedure type), type of intervention, and reported outcomes. Specifically, the “type of intervention” category captured detailed information on the telecommunication technologies used (e.g., video conferencing, remote imaging), and the dental specialties addressed (such as orthodontics, periodontics, etc.). Any discrepancies in data extraction were addressed through discussion between reviewers, with unresolved cases being adjudicated by a third independent evaluator (PO).

### Bias assessment

Two reviewers (AT and KG) separately assessed the risk of bias using the Newcastle-Ottawa scale (NOS), and disagreements were discussed [ 13 ]. If disagreements could not be resolved, a third reviewer interfered. NOS examined three key aspects: (1) participant selection procedures, (2) between-group comparability, and (3) outcome measurement validity. The scale employed a star-based rating system (maximum 9 stars) where individual criteria within each domain are assessed, with more stars awarded for lower bias risk. The cumulative star rating across all domains provided a comprehensive study quality evaluation, where higher total scores reflect more rigorous methodology.

### Statistical Analysis

**Figure 1:**
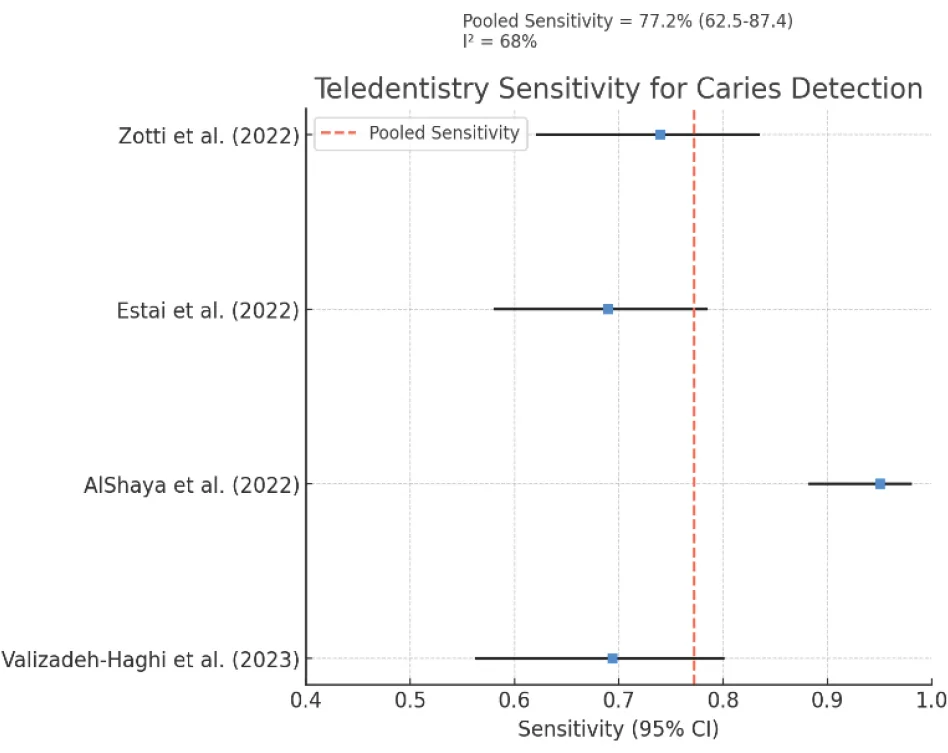
Forest plots of included studies

Quantitative evaluation and meta-analysis of the four included studies were conducted using a systematic review software. Results from the meta-analysis were graphically represented through forest plots, which clearly display the summary effect sizes along with their 95% confidence intervals (Figure 1). This facilitated visualization of both the precision and variability of the pooled estimates. 

Heterogeneity among studies was assessed using the I² statistic, which estimated the percentage of variation across studies due to heterogeneity rather than chance. Significant heterogeneity was observed (I² = 68% for sensitivity and 92% for specificity), likely attributable to differences in diagnostic criteria, imaging protocols, and examiner experience. Consequently, a random-effects model was employed to account for this expected variability.

Sensitivity analyses were performed by sequentially excluding each study to assess its impact on the pooled estimates, with stability indicated by minimal changes in sensitivity (Δ sensitivity <5%). Due to the limited number of included studies, subgroup analyses were not conducted, but are recommended for future research with larger sample sizes.

Publication bias was not formally assessed (e.g., via funnel plots or Egger’s test) given the small number of studies included, which limits the reliability of such tests. The importance of evaluating publication bias is high, which suggests its inclusion in future reviews with more extensive datasets. 

## Results

### Study Characteristics

**Figure 2:**
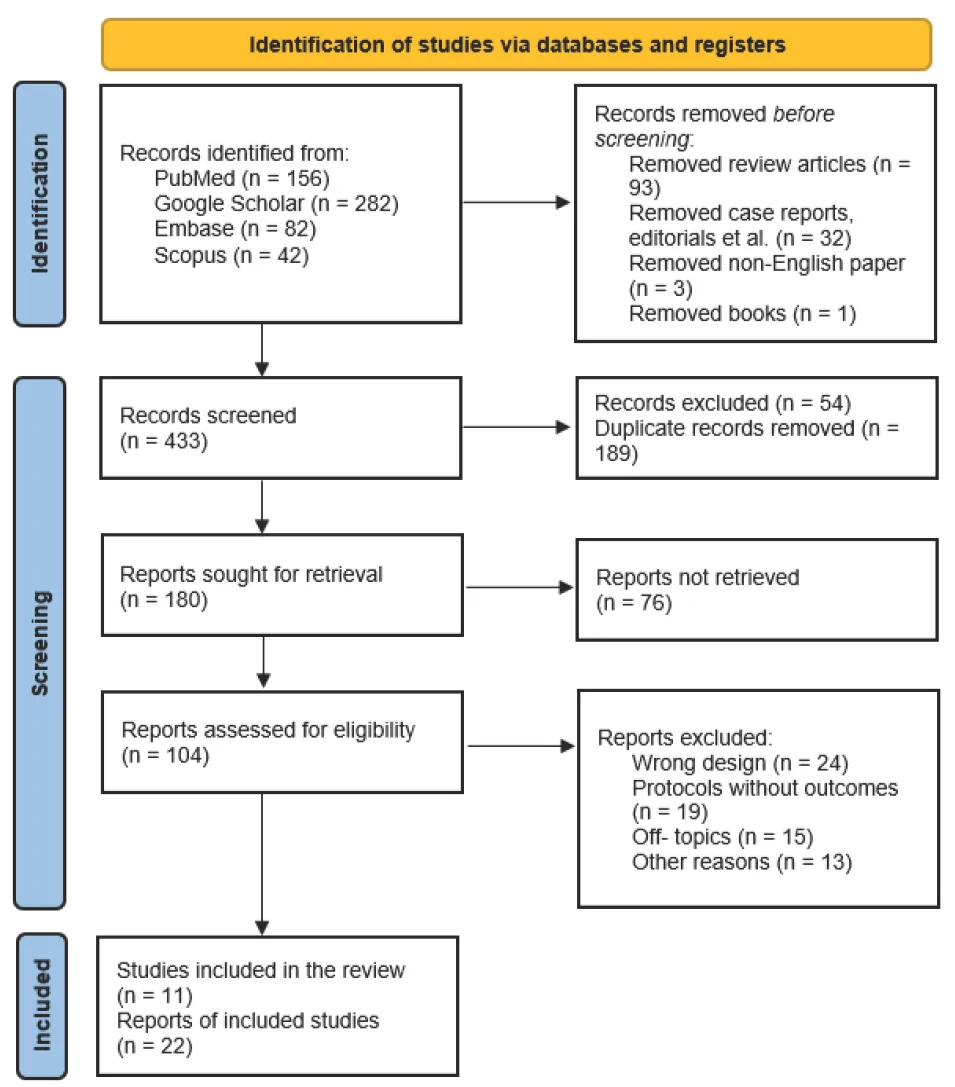
The study's methodology based on PRISMA guidelines

The literature search initially identified 562 potentially relevant studies. The identified articles then underwent a rigorous selection process using established inclusion/ exclusion criteria. First, duplicate records were eliminated using the Rayyan software, leaving only unique publications. These were initially evaluated by reviewing titles and abstracts, during which clearly irrelevant studies or those outside the study's scope were excluded. The remaining articles then progressed to a comprehensive full-text evaluation against the inclusion criteria. Through this multi-stage screening approach, involving deduplication, title/abstract screening, and full-text assessment, 11 studies [ 14 - 24 ] ultimately qualified for inclusion in the systematic review (Figure 2). 

The included studies were published between 2020 and 2024 and spanned multiple geographic locations, including Italy [ 14 ], Australia [ 15 ], Saudi Arabia [ 16 ], Iran [ 17 - 18 ], the United Kingdom [ 19 , 22 ], India [ 20 ], the United States [ 21 ], Brazil [ 23 ], and Fiji [ 24 ], demonstrating wide global representation and enhancing the applicability of results across diverse populations. Moreover, sample sizes varied considerably (6 to 364 participants), offering insights into teledentistry's effectiveness across different demographic scales.

Studies encompassed participants ranging from infants (<1 year) to seniors (≥65 years), thus demonstrating teledentistry's potential utility across all age groups. Furthermore, gender distribution varied among studies, with several reporting a predominance of male participants. While this demographic diversity and variation in sample sizes were well recognized, their potential impact on diagnostic accuracy was not formally analyzed through subgroup methods due to the limited number of included studies. Therefore, such analyses are recommended for future research to better understand these influences. Table 2 shows a summary of the included studies.

**Table 2:** Characteristics of reviewed studies

Author and Location	Purpose	Area	Gold Standard Examination	Tele-dentistry Examination	Sample Size and Age	Study Design	Results
Zotti *et al*. [14], Italy	Evaluate the effectiveness and accuracy of teledentistry in detecting dental caries compared to clinical examination	Restorative dentistry	Visual examination (ICDAS II)	5 intraoral images per patient using smartphones	Forty-three patients	Cross-sectional	The sensitivity of teledentistry was 74.0%, and the specificity was 99.1%
Estai *et al*. [15], Australia	Compare the use of intraoral photographs with the unaided visual dental examination as a means of dental caries detection in children	Restorative dentistry	Visual examination (DFT)	5 intraoral images per patient using smartphones	138 children aged 4 to 14 years old	Cross-sectional	The sensitivity and specificity of the photographic method were 58-80 percent and 99. 7-99.9 percent, respectively
AlShaya *et al*. [16], Saudi Arabia	Assess the accuracy of teledentistry in caries detection compared with the clinical dental examination	Restorative dentistry	Visual examination (DMFT)	5 intraoral images per patient using smartphones	95 children aged 5 to 10 years old	Cross-sectional	In primary teeth, tele-dentistry showed 95% sensitivity and 94.3% specificity
Valizadeh-Haghi *et al*. [17], Iran	Compare the use of smartphone photography with the face-to-face examination in the evaluation of anterior composite restoration	Restorative dentistry	Visual examination (FDI)	6 intraoral images per patient using smartphones	6 patients	Observational study	The sensitivity and specificity of the smartphone photography method were 69.35% and 48.72% respectively
Niknam *et al*. [18], Iran	Evaluate the usability and reliability of a teledentistry tool for the remote diagnosis of oral lesions	Oral medicine and Periodontology	Visual examination	262 intraoral images using smartphones	109 patients with an average age of 47±19.46 years	Cross-sectional	The reliability test showed that the web-based teleconsultation system performed significantly well, and significant performance was seen (p&gt; 0.05)
Cronin *et al*. [19], United Kingdom	Assess the acceptability of patients and clinicians towards teleconsultation in oral and maxillofacial surgery compared with an expected face-to-face assessment	Oral and maxillofacial surgery	Face-to-face appointment and assessment	420 telephone consultations	340 patients	Prospective study	A total of 59.1% of patients expressed a strong preference for teleconsultation and its diagnostic accuracy was 43.5%
Sathe *et al*. [20], India	Assess the attitude, awareness and knowledge of dental practitioners towards the use of digital radiography	Oral and maxillofacial radiology	Dental radiographs	10 Digital radiographs	350 responders	Cross-sectional	79.71% of participants reported preferred using digital radiography
Homsi *et al*. [21], United States	Evaluate the acceptance of patients and their orthodontists on the use of different modes of communication through Tele-orthodontics	Orthodontics	Visual examination	Online survey using HTML and QR code	364 patients aged from 18–65 years	Cross-sectional	Overall satisfaction with using Tele-orthodontics was recorded at 92%
Viswanathan *et al*. [22], United Kingdom	Assess the effectiveness of the service provided by pediatric dental consultants	Pediatric Dentistry	Visual examination	Telephone consultation	215 patients aged from <1 to 16 years old	Retrospective study	This study showed a 97% response rate
Paixão *et al*. [23], Brazil	Evaluate the Telehealth Brazil Networks Program’s dental teleconsulting to elucidate the prevalent questions in endodontics	Endodontics	Visual examination	3920 teleconsultations	3920 patients	Cross-sectional and exploratory study	Endodontics was the field with the sixth-highest demand for teleconsultations (7.4%)
Lakshmikantha *et al*. [24], Fiji	Investigate the feasibility and effectiveness of teledentistry to enhance access to prosthodontic care	Prosthodontics	Visual examination	Teledentistry platform incorporating real-time video conferencing, digital impression-taking, and remote monitoring	126 participants	Cross-sectional	Teledentistry showed promise in pilot testing, with high user satisfaction reported

Out of the eleven included studies, four focused on restorative dentistry [ 14 - 17 ], while one study was conducted in each of the following other fields: oral medicine and periodontology [ 18 ], oral and maxillofacial surgery (OMFS) [ 19 ], oral and maxillofacial radiology [ 20 ], orthodontics [ 21 ], pediatric dentistry [ 22 ], endodontics [ 23 ], and prosthodontics [ 24 ]. Moreover, most of the included studies employed a cross- sectional design. The studies exhibited variability in teledentistry equipment. Intraoral cameras and smartphones were used for dental imaging in five of the eleven studies [ 14 - 18 ], while three studies relied on telecommunication methods [ 19 , 22 - 23 ], one utilized dental radiography [ 20 ], and one employed video conferencing [ 24 ]. In other words, all studies adopted an asynchronous approach, except for one that used synchronous communication via videoconferencing [ 24 ].

The most frequently reported gold standard was traditional visual examination, although the specific methods and tools varied across studies. Equipment used included light sources, sterilized exploration kits, probes, air syringes, mirrors, explorers, and palpation. Ten studies employed visual clinical examination for dental caries and oral pathology assessment and evaluating orthodontic, prosthodontic, endodontic, and surgical needs of the patient [ 14 - 19 , 21 - 24 ]. Meanwhile, one study used only dental radiographs, including panoramic and periapical images, to assess dental condition [ 20 ]. Although the gold standard method varied in this research from others, the reported results were similar.

Examiner qualifications differed across studies, ranging from general dentists to dental practitioners. Some studies utilized dental practitioners for remote and gold-standard examinations [ 14 , 17 - 18 , 20 - 22 ], while others simply mentioned "dental practitioners" without specifying their qualifications or experience levels [ 15 - 16 , 19 , 23 - 24 ]. Furthermore, in all studies, assessors were independent across telecommunication and in-person conditions to minimize potential bias and ensure objective evaluation.

The meta-analysis in this study evaluated the diagnostic accuracy of teledentistry compared to in-person examinations, focusing on four studies that reported sensitivity and specificity for caries detection [ 14 - 17 ]. Pooled results showed a moderate sensitivity of 77.2% (95% CI: 62.5–87.4%), indicating that teledentistry can detect most true caries cases but may miss early lesions. They also showed a high specificity of 89.1% (95% CI: 70.5–96.5%), indicating a strong ability to rule out non-caries cases, though with variability across studies.

Heterogeneity was significant (I² = 68% for sensitivity, 92% for specificity), likely due to varied diagnostic thresholds (ICDAS II≥3 vs. DMFT≥4), differing smartphone camera specifications (8MP vs. 12MP), and inconsistent lighting conditions across studies. Moreover, sensitivity analyses showed stability of estimates (Δ sensitivity <5%) when excluding any single study.

While the consistent use of smartphone photography reduced one potential source of heterogeneity, persistent variability was attributed to unstandardized implementation factors. These findings underscore the need for minimum technical standards for teledentistry photography (e.g., resolution, lighting), standardized diagnostic criteria in future studies, and blinded examiner protocols to minimize operator bias.

Findings align with the qualitative review, confirming teledentistry’s viability, particularly in restorative and pediatric dentistry, while highlighting limitations in image quality and examiner training. For instance, Valizadeh-Haghi *et al*. [ 17 ] found that smartphone photography yielded relatively low sensitivity (69.35%) and specificity (48.72%), likely due to inadequate image resolution and inconsistent lighting conditions. Similarly, Cronin *et al*. [ 19 ] reported a diagnostic accuracy of only 43.5% in teleconsultations, suggesting that the absence of formal calibration or standardized training among assessors may contribute to suboptimal performance. By contrast, AlShaya *et al*. [ 16 ] demonstrated high sensitivity (95%) and specificity (94.3%) in primary teeth detection, indicating that when imaging conditions are optimized and assessors are likely more experienced, diagnostic accuracy can be considerably improved. These findings emphasize the need for standardized imaging protocols and structured assessor training to enhance the reliability of teledentistry applications across diverse clinical settings.

Overall, the reviewed studies demonstrated comparable diagnostic accuracy between teledentistry assessments and in-person clinical examinations for both primary and permanent dentition. This finding indicates its viability as an effective alternative to traditional diagnostic approaches. Additionally, this outcome highlights teledentistry's critical role in expanding access to dental care for remote or underserved populations with limited availability of traditional services.

### Risk of Bias Assessment 

All studies achieved a score between 6 and 9 stars on NOS, indicating overall moderate to high methodological quality. Specifically, two studies (Valizadeh-Haghi *et al*. [ 17 ], Sathe *et al*. [ 20 ]) received 6 stars and were rated as moderate quality, while the remaining nine studies achieved between 7 and 9 stars, qualifying as good quality. The highest possible score (9 stars) was obtained by AlShaya *et al*. [ 16 ], Homsi *et al*. [ 21 ], and Paixão *et al*. [ 23 ], reflecting strong performance across selection, comparability, and outcome/exposure domains. In contrast, the weaker aspects in several studies were most often found in the comparability domain and the outcome/ exposure domain. This distribution suggested that while the overall evidence base is robust, improvements in study design could further enhance the quality of future teledentistry research. The details can be seen in Table 3. 

**Table 3:** Newcastle-Ottawa scale (NOS) Risk of Bias Assessment of included studies

Study	Selection (Max 4★)	Comparability (Max 2★)	Outcome/Exposure (Max 3★)	Total (Max 9★)	Quality
Zotti *et al*. [14]	★★★✗	★★	★★★	8★	Good
Estai *et al*. [15]	★★★★	★★	★★✗	8★	Good
Al Shaya *et al*. [16]	★★★★	★★	★★★	9★	Good
Valizadeh-Haghi *et al*. [17]	★★★✗	★✗	★★✗	6★	Moderate
Niknam *et al*. [18]	★★★✗	★★	★★★	8★	Good
Cronin *et al*. [19]	★★★✗	★★	★★✗	7★	Good
Sathe *et al*. [20]	★★★✗	★✗	★★✗	6★	Moderate
Homsi *et al*. [21]	★★★★	★★	★★★	9★	Good
Viswanathan *et al*. [22]	★★★✗	★★	★★✗	7★	Good
Paixão *et al*. [23]	★★★★	★★	★★★	9★	Good
Lakshmikantha *et al*. [24]	★★★✗	★★	★★★	8★	Good

## Discussion

Teledentistry integrates insights from various scientific disciplines, resulting in its classification into two primary branches: synchronous and asynchronous [ 25 - 27 ]. Synchronous teledentistry is characterized by real-time interaction between the patient and the dentist. Essentially, this involves direct communication and interaction through digital tools such as cameras, speakers, and microphones, all facilitated by the internet. For example, a dentist may conduct a real-time video examination of a patient’s oral lesion using an intraoral camera, allowing for immediate diagnosis and treatment planning. This method is particularly useful for emergency consultations or follow-up visits, as it enables instant feedback. However, it requires stable internet connectivity and scheduling coordination between parties [ 28 - 29 ].

Meanwhile, asynchronous teledentistry begins with the collection of patient data. This information is then sent to the dentist for review at a later time, and the dentist's recommendations are subsequently communicated back to the patient. For instance, a common application of this method is in rural screening programs, where community health workers upload dental images for remote specialists to assess and provide recommendations within 24-48 hours. While this approach offers flexibility and reduces the need for simultaneous availability, delays in response time may limit its use for urgent cases [ 28 - 29 ]. Both types of teledentistry are now effectively being used in multiple dental specialties [ 1 , 29 ], which is discussed below. 

In the field of restorative dentistry, dentists routinely employ visual examinations and radiography in clinics for immediate caries detection and intervention. Nonetheless, direct clinical examinations can be costly and sometimes impractical, particularly for individuals in remote or underserved areas. In such cases, teledentistry, utilizing high-quality cameras or smartphones to capture dental photographs for remote assessment, offers a viable alternative. However, it's crucial to note that capturing high-resolution images suitable for accurate caries assessment requires both theoretical knowledge and practical training for dentists [ 30 ].

Zotti *et al*. [ 14 ] conducted a study in which 10 photographs per patient were taken from five different angl–es using smartphones and evaluated by an experienced dentist. Subsequently, a second dentist performed clinical examinations, classifying caries using the ICDAS II system. The findings revealed no significant difference between teledentistry and traditional clinical observation. Notably, these results align with other research, which has employed various caries classification systems like DMF [ 31 ], DFT [ 15 ], DMFT [ 16 ], and DFS [ 32 ].

While several studies have highlighted the effectiveness of teledentistry in diagnosing dental caries [ 14 - 17 ], a contrasting study by AlShaya *et al*. [ 33 ] in 2020, conducted on 57 children aged 6-12 years, indicated that teledentistry could not replace the conventional gold standard for caries detection. Specifically, a pediatric dentist, after reviewing smartphone photographs; two extraoral (anterior and lateral) and five intraoral (anterior closed mouth, upper occlusal, lower occlusal, right lateral, left lateral), could only reliably diagnose initial caries. To sum up, this study proved that radiographic examination is essential for valid caries detection. Moreover, this research indicated that teledentistry based on photographic images of patients' teeth is not reliable. It should be noted that this research utilized the WHO standard forms for caries assessment [ 33 ]. 

The observed discrepancies between studies likely reflect three key factors: imaging protocols (7 versus 10 images per patient), caries classification systems (WHO versus ICDAS II), and patient populations (children versus mixed-age groups) [ 14 , 33 ]. While teledentistry using high-quality photographs offers a practical solution for remote assessment, evidence suggests it cannot fully replace conventional clinical examinations. Therefore, further research is needed to define the complementary role of teledentistry alongside traditional diagnostic methods in restorative dentistry [ 14 - 17 , 33 ].

The advancement of teledentistry in periodontology has spurred the development of the term "Tele-periodontics" [ 34 ]. This innovative approach involves a general dentist, collecting information regarding the patient's oral health and medical history along with detailed intraoral photographs for consultation with a periodontist specialist. Real-time online consultations, while requiring a robust internet connection, allow the general dentist to perform procedures like probing as directed by the specialist, who can observe the results remotely [ 34 ]. This collaborative approach enables specialists to plan subsequent treatment steps effectively based on examination findings. Beyond diagnosis and treatment of periodontal issues, Tele-periodontics also proves valuable for prescribing medication for acute problems, patient education, and ongoing follow-up care [ 34 ].

With the emergence of Tele-periodontics, studies have presented conflicting findings regarding its effectiveness [ 35 - 36 ]. For instance, a study by Penmetsa *et al*. [ 35 ] surveyed 350 oral and dental surgeons using 29 short-answer questions to assess their knowledge, attitude, and perceived performance of Tele-periodontics. The results indicated that a significant majority (80.57%) were familiar with teledentistry techniques, and a strong majority (78%) agreed with its clinical application. Furthermore, a considerable proportion believed in its diagnostic capabilities for periodontal conditions (72.29%) and its positive impact on patient follow-up (70.29%) [ 35 ].

In contrast, Sritam *et al*. [ 36 ] presented contrasting results from their survey of 120 oral and dental surgeons, which used a similar design with 27 short-answer questions on Tele-periodontics knowledge, attitude, and performance. Their findings indicated that a substantial majority (80.8%) were unfamiliar with teledentistry, and even more (81.7%) disagreed with its clinical use. Moreover, a similar percentage (80.8%) doubted its diagnostic utility for periodontal conditions, and again, a larger proportion (81.7%) questioned its effectiveness in patient follow-up [ 36 ]. 

The conflicting findings between Penmetsa *et al*. [ 35 ] and Sritam *et al*. [ 36 ] may stem from differences in sample size, survey design, and participants’ exposure to Tele-periodontics training. Penmetsa *et al*. [ 35 ] surveyed more clinicians and may have included those with greater familiarity or recent education in teledentistry, while Sritam *et al*.’s [ 36 ] smaller sample may reflect limited awareness. These factors highlight the need for standardized methodologies and larger, multi-center studies to more accurately assess the effectiveness and acceptance of Tele-periodontics [ 35 - 36 ].

Frequent oral and maxillofacial surgery-related conditions, including temporomandibular joint disorder, salivary gland disorders, head and neck cancer, orthognathic problems, and facial and jaw trauma, traditionally necessitate in-person consultations. However, recent research highlights the growing potential of teledentistry within OMFS [ 37 - 38 ]. For instance, a study conducted during the COVID-19 pandemic examined the use of telephone consultations for patients requiring OMFS. This research revealed that accurate diagnoses and appropriate treatment plans were successfully achieved in an impressive 98.7% of 151 cases that consulted OMFS surgeons. Hence, the results of this research emphasized eliminating the need for subsequent face-to-face appointments by using teledentistry [ 39 ].

In contrast, another study from the same pandemic period exploring the effectiveness of teledentistry for OMFS patients presented different outcomes. Although 59.1% of patients expressed a preference for teledentistry over traditional consultations, accurate diagnoses and effective treatment solutions were reached in only 43.5% of cases. This disparity was attributed to the inherent limitations of telephone consultations when clinical examinations were not possible. This finding highlights the limitations of relying solely on telephone consultations for diagnosing and managing oral conditions that may require surgical intervention [ 23 ].

The conflicting observations in studies regarding OMFS teledentistry likely reflect differences in consultation methods, patient populations, and condition types [ 23 , 39 ]. High accuracy in one study [ 39 ] may result from structured telephone consultations with cases suitable for remote assessment, while lower accuracy in another [ 23 ] reflects reliance on telephone-only evaluations for a broader patient group. These discrepancies highlight the need for standardized protocols and condition-specific criteria to clarify when teledentistry can be reliably applied in OMFS [ 23 , 39 ]. 

In addition to the two aforementioned studies, a more recent research sheds light on teledentistry's positive impact on post-operative patient monitoring. In this research, patients who had undergone surgery for conditions such as acute dental abscesses, neoplastic lesions, tooth fractures, oroantral communication, cystic lesions, and impacted third molars were instructed to photograph their mouths on postoperative days 3, 7, and 14 and send the images to their surgeons. By comparing these post-operative photos with pre-operative records, surgeons were able to effectively manage and guide patients through the critical early post-surgical period and during subsequent follow-up care. Ultimately, this study suggests that while concerns may persist about tele-dentistry’s role in pre-surgical diagnosis and management, it holds significant promise as a valuable tool for post-operative care [ 9 ].

Teleradiology first emerged in Mumbai, India, in 1996, pioneering the remote interpretation of radiographic images from anywhere in the world [ 40 ]. Recognizing its significant impact, teleradiology has quickly become particularly valuable in addressing radiologist shortages during off-hours and overnight shifts. Beyond these broader applications, teleradiology offers dentists a powerful tool for interpreting panoramic radiographs, aiding in the diagnosis of lesions, caries, and temporomandibular joint disorders from a distance. Therefore, this faster diagnostic capability for dental issues reduces delays in treatment planning, saving both time and resources [ 40 ].

For teleradiology, various radiographic images, both intraoral and extraoral, including CT, CBCT, ultrasound, MRI, and nuclear medicine scans, must first be digitized. Today, DICOM is widely employed as the standard to secure data transfer while maintaining high image quality. However, the technique still faces challenges, including the need for high-resolution monitors and robust data security. To address these challenges, organizations have proposed regulations like ISO17799, focusing on the protection and organization of sensitive health data [ 40 ].

Despite these limitations, teleradiology's inherent advantages, such as increased availability, accessibility, reduced travel time, and cost-effectiveness. Nevertheless, a significant concern lies in the potential for interpretations and reports generated by unqualified individuals, often referred to as "ghost radiologists." The pressure to prioritize speed in reporting over accuracy and quality can also be problematic. Furthermore, some teleradiologists prefer remote reporting without direct patient interaction, adding another layer of complexity. Consequently, while teleradiology offers numerous benefits, these challenges have limited its widespread adoption beyond specific studies and certain countries. Addressing these issues is crucial for realizing its full potential [ 40 ].

Orthodontic problems affecting teeth and jaws place a considerable financial, psychological, and aesthetic burden on patients. Early diagnosis by a dentist can significantly reduce the severity of these issues, along with treatment costs and duration. Therefore, integrating teledentistry with modern orthodontics allows dentists to monitor patients remotely, rapidly assess dental and jaw concerns, and develop appropriate treatment plans [ 10 , 41 ].

Compared to traditional methods, teledentistry in orthodontics enables dentists to treat more patients concurrently without sacrificing care quality. In fact, this technique allows regular monitoring, more frequent and accurate assessments of a patient's dental condition, remote treatment instructions, and adjustments to subsequent treatment steps. For instance, teledentistry facilitates the early detection of malocclusion recurrence, even with minor tooth movement. Furthermore, dentists can remotely adjust the placement of intermaxillary elastics within a patient's treatment plan based on their current condition [ 41 - 42 ].

Orthodontic treatments are often lengthy, requiring years of dental supervision. Teledentistry offers the advantage of remote patient monitoring, which is particularly beneficial for individuals who travel frequently or live abroad [ 41 ].

Removable appliances are often recommended in orthodontics, especially for children. However, challenges such as speech and eating difficulties, and mouth sores, can lead to patients discontinuing their use. Effective and consistent communication, along with dental encouragement, is crucial during this phase to achieve a successful treatment outcome. Therefore, it is reported that teledentistry's ability to improve patient well-being and establish efficient dentist-patient communication is a key factor driving its increased adoption [ 43 ].

Despite these benefits, in-person clinic visits remain necessary in some cases, particularly with fixed orthodontics. This is due to the increased risk of damage to fixed appliances or the presence of mouth sores after appointments which make remote treatment impractical. Hence, teledentistry in orthodontics is primarily recommended for patient monitoring and initial problem diagnosis [ 21 ].

Children and adolescents are a key focus for early diagnosis and prevention of oral and dental diseases. Despite ongoing efforts to provide dental health services to this age group, significant barriers to accessing treatment remain due to geographical, social, and economic factors. This limited access necessitates cost-effective and sustainable solutions to improve children's dental care [ 37 ].

One crucial method for enhancing overall oral health within society is through effective education during childhood. Children today are adept and enthusiastic users of digital technology, making mobile-based oral health education programs a valuable tool for educating them, even in remote areas [ 37 ].

Beyond preventative education, teledentistry is increasingly being utilized to deliver services to pediatric patients in underserved regions. For example, children experiencing delayed tooth eruption may require consultation with a pediatric dentist. In such cases, an initial video call to assess the oral cavity can effectively determine if a more comprehensive, in-person examination is needed. Additionally, common developmental dental issues in children, such as natal teeth and eruption cysts, can be monitored and managed remotely through teledentistry [ 32 ].

While teledentistry is recognized as a potentially beneficial approach for children's dental care, numerous clinical studies have been conducted to validate this and assess its effectiveness [ 5 , 33 ]. For instance, Kanani *et al*. [ 5 ] investigated the feasibility of using intraoral cameras and teledentistry to screen dental conditions, including early childhood caries, in 50 preschoolers aged 4 to 6 years. Their findings showed no statistically significant difference between diagnoses made using intraoral cameras and those from traditional in-person examinations for early childhood caries. In the end, this research proposed that teledentistry offers a suitable and cost-effective alternative for caries screening in preschool children [ 5 ].

In comparison, Viswanathan *et al*. [ 22 ] explored the effectiveness of teledentistry for pediatric and orthodontic consultations in 215 children with lip and palate clefts. This research revealed that approximately 86% of these patients could be effectively managed through remote consultations and did not require urgent referral to a dentist. Consequently, this study highlighted teledentistry's potential to significantly reduce costs and save time for children with cleft lip and palate [ 22 ].

In endodontics, the initial step for accurate diagnosis and effective treatment planning is assessing the vitality of the dental pulp and surrounding periodontal tissues. However, determining pulp vitality relies on clinical tests, notably thermal tests, making tele-dentistry less efficient in this area. Furthermore, a dentist's diagnosis in the teledentistry technique heavily depends on patient-provided information, including symptom descriptions and radiographic images. Consequently, incomplete or inaccurate patient reporting can substantially reduce diagnostic accuracy and affect subsequent treatment planning [ 44 ].

Regarding the use of teledentistry in endodontics, studies indicate that successful diagnosis and treatment planning are strongly linked to the dentist's level of expertise. Given the complex nature of endodontic problems, specialist consultation and case review by an endodontist can significantly improve treatment outcomes compared to a general dentist. Therefore, it is generally recommended to limit the application of teledentistry in endodontics and prioritize in-person dental visits when ever possible [ 23 ].

Teledentistry in prosthodontics not only offers benefits that go beyond initial consultations, diagnoses, and treatment planning (based on patient information and radiographs) but also is a valuable tool for managing patients during emergency situations [ 45 ]. For instance, when patients with removable dentures (partial or complete) experience a fracture or poor fit, dentists can use teledentistry to remotely assess the problem. Dentists can then advise the patient to discontinue denture use until an in-person appointment can be scheduled [ 45 ].

Conversely, for patients with fixed prostheses experiencing a loosened or detached crown on an abutment tooth, teledentistry allows dentists to guide them. In other words, patients are instructed to remove the crown through video calls, and if the abutment tooth is sensitive, dentists can prescribe silver diamine fluoride or casein phosphopeptide amorphous calcium phosphate. Similarly, in cases where a patient with a bridge loses a crown from just one abutment tooth, video consultations enable dentists to demonstrate how to maintain cleanliness under the bridge using specialized floss until an in-person visit is possible [ 45 - 46 ].

Beyond tooth-supported prostheses, teledentistry is also feasible for implant-supported denture emergencies. Like their tooth-supported counterparts, implant prostheses can also encounter issues such as peri-implantitis and screw loosening, necessitating dental consultation. In peri-implantitis scenarios, teledentistry has been shown to facilitate patient management, potentially including the remote prescription of antibiotics [ 45 - 46 ]. On the other hand, while loose implant or abutment screws can sometimes be tightened remotely with guidance, broken screws still require in-person intervention. Furthermore, if a screw completely detaches, dentists can even supervise patients in obtaining a temporary restoration from online retailers like Amazon. Therefore, teledentistry emerges as a particularly useful and effective approach in prosthodontics, especially when addressing emergency situations [ 45 - 46 ].

Despite the numerous advantages of teledentistry, its widespread adoption is still limited by various challenges. These obstacles can be broadly categorized into personnel-related and technological factors [ 47 ].

Personnel factors include the significant costs associated with implementing teledentistry, limited financial profitability, and, importantly, patient reluctance to embrace remote consultations. Research indicates that patients often have less confidence in diagnoses made re-motely compared to in-person examinations. Moreover, the complexity of using electronic devices for communication can deter some individuals, especially the elderly, further contributing to patient rejection [ 47 - 48 ].

Technological factors also present significant obstacles. These include the need for readily accessible software and hardware, along with adequate user training. Notably, many individuals in underserved areas lack access to the necessary technology or reliable internet for teledentistry. Even with sufficient equipment and connectivity, technical issues can still arise during consultations, highlighting the need for robust and readily available online support. Given the high cost of dedicated support teams, integrating training on teledentistry equipment and tools into dental school curricula is increasingly recommended [ 47 , 49 - 50 ].

Artificial intelligence (AI) is among the emerging technologies that have rapidly gained prominence in dentistry. Today, we are seeing AI models applied to interpreting patient radiographs by analyzing radiopacity differences, assessing cavity depth, and suggesting methods to restore dental caries. It is also used to identify pathological lesions in radiographic images and, most importantly, to combine radiographic, photographic, demographic, and patients' overall health information to develop appropriate treatment plans [ 51 ].

Despite these advances, implementing AI in dental practice presents several technical challenges. These include the need for large, high-quality datasets, potential biases in algorithmic predictions, and difficulties integrating AI systems into existing clinical workflows. Current limitations also persist, such as reduced diagnostic accuracy in complex cases, lack of transparency in AI decision-making, ethical issues, and the risk of over-reliance on automated recommendations [ 52 ].

Given AI’s capabilities in various dental specialties, some studies have reported that combining it with teledentistry to provide remote diagnoses and treatment plans, using patient-provided information, can, in certain cases, outperform a trained dentist [ 52 - 53 ]. Essentially, the use of AI models enables patients to submit their information to the system and receive dental consultations at any time, day or night, which also saves dentists' valuable time. However, it should be noted that due to the inherent uncertainty in AI models providing perfectly accurate diagnoses and treatment plans, the results presented to patients must always be verified by a dentist [ 53 ].

In addition to the aforementioned limitations of AI, the scarcity of high-quality research limits the ability to draw definitive conclusions about its diagnostic performance and clinical utility in remote dental care settings. This highlights the urgent need for rigorous clinical evaluation, including controlled trials, assessment of patient outcomes, cost-effectiveness analyses, and adherence to regulatory standards. Addressing these challenges will provide a more realistic understanding of AI’s potential and safe application in dentistry [ 52 - 53 ].

## Conclusion

Teledentistry is increasingly transforming dental care by enhancing access, efficiency, and patient convenience across multiple specialties. It facilitates remote consultations, diagnostics, and treatment planning. Moreover, this approach offers significant advantages in bridging geographical gaps, reducing costs, and ensuring continuity of care in crisis situations, such as pandemics. Despite these benefits, several limitations persist, including technological constraints, data privacy and security concerns, regulatory challenges, and variability in practitioner and patient acceptance. Potential solutions include developing robust telecommunication infrastructure, user-friendly platforms, standardized regulatory frameworks, comprehensive data protection protocols, and targeted training programs for both clinicians and patients. Therefore, addressing these challenges through systematic research and evidence-based strategies will be essential for the sustainable and effective integration of teledentistry into routine oral healthcare. 
